# The Angiopoietin-1 Variant COMP-Ang1 Enhances BMP2-Induced Bone Regeneration with Recruiting Pericytes in Critical Sized Calvarial Defects

**DOI:** 10.1371/journal.pone.0140502

**Published:** 2015-10-14

**Authors:** Hyuck Choi, Byung-Chul Jeong, Sung-Woong Hur, Jung-Woo Kim, Keun-Bae Lee, Jeong-Tae Koh

**Affiliations:** 1 Department of Pharmacology and Dental Therapeutics, Research Center for Biomineralization Disorders, School of Dentistry, Chonnam National University, Gwangju, Republic of Korea; 2 Department of Orthopedic Surgery, Chonnam National University Medical School and Hospital, Gwangju, Republic of Korea; Tokyo Medical and Dental University, JAPAN

## Abstract

Craniofacial bone defects are observed in a variety of clinical situations, and their reconstructions require coordinated coupling between angiogenesis and osteogenesis. In this study, we explored the effects of cartilage oligomeric matrix protein-angiopoietin 1 (COMP-Ang1), a synthetic and soluble variant of angiopoietin 1, on bone morphogenetic protein 2 (BMP2)-induced cranial bone regeneration, and recruitment and osteogenic differentiation of perivascular pericytes. A critical-size calvarial defect was created in the C57BL/6 mouse and COMP-Ang1 and/or BMP2 proteins were delivered into the defects with absorbable collagen sponges. After 3 weeks, bone regeneration was evaluated using micro-computed tomography and histologic examination. Pericyte recruitment into the defects was examined using immunofluorescence staining with anti-NG2 and anti-CD31 antibodies. *In vitro* recruitment and osteoblastic differentiation of pericyte cells were assessed with Boyden chamber assay, staining of calcified nodules, RT-PCR and Western blot analyses. Combined administration of COMP-Ang1 and BMP2 synergistically enhanced bone repair along with the increased population of CD31 (an endothelial cell marker) and NG2 (a specific marker of pericyte) positive cells. *In vitro* cultures of pericytes consistently showed that pericyte infiltration into the membrane pore of Boyden chamber was more enhanced by the combination treatment. In addition, the combination further increased the osteoblast-specific gene expression, including bone sialoprotein (BSP), osteocalcin (OCN) and osterix (OSX), phosphorylation of Smad/1/5/8, and mineralized nodule formation. COMP-Ang1 can enhance BMP2-induced cranial bone regeneration with increased pericyte recruitment. Combined delivery of the proteins might be a therapeutic strategy to repair cranial bone damage.

## Introduction

Repair of bone defects requires a coordinated coupling between osteogenesis and angiogenesis for regeneration [1]. It involves a multistep process that includes migration, proliferation, and differentiation of several types of cells such as endothelial cells, fibroblasts, osteoblasts, osteoclasts and pericytes within the bone microenvironment [2, 3]. Angiogenesis has an impact on bone formation since oxygen, nutrients, osteoinductive factors and stem cells are supplied into the defect area through the blood stream. In addition, the formation of vascular wall contributes to migration of osteoblast progenitor cells such as pericytes into target site [4].

Pericytes are specialized cells that wrap around the endothelial cells of capillaries and venules, and play critical roles in various physiological contexts, including support of vascular structure and function, initiation of vessel sprouting and stabilization of vessel. Mesenchymal stem cells (MSCs) and pericyte progenitors are both perivascular cells with similar multipotent properties regardless of tissue of origin; they are shown to be capable of differentiating into osteoblasts, chondrocytes, adipocytes and fibroblasts under special stimulations [5, 6]. Moreover, pericytes have come under increasing scrutiny as possible osteogenic precursors because they express the bone matrix protein such as BSP and OCN. Thus, a growing interest exists in the recruitment, proliferation, and osteoblastic differentiation of pericytes for therapeutic bone regeneration. Proliferation and recruitment of pericytes are under the influence of angiogenic growth factors, which are secreted from surrounding cells in the microenvironment such as endothelial cells. For example, endothelial-derived PDGF-BB and HB-EGF coordinately regulate pericyte recruitment during vasculogenic tube assembly and stabilization [7, 8]. VEGF-A and angiopoietin-1 (Ang-1) also act on pericytes in an autocrine and paracrine manner to stimulate their proliferation and migration with activation of their receptors [9, 10].

Bone morphogenetic proteins (BMPs) are well characterized as the most potent osteoinductive factors to differentiate MSCs into osteoblasts and play a critical role in osteogenesis, fracture repair and bone regeneration [11]. Since recombinant BMP2 became available, many animal studies have been performed to examine the induction of bone formation and repair of bony defects following implantation of BMP2 [12]. Combined treatment with angiogenic factors and BMPs has been considered for the improvement of reconstruction of large craniofacial defects. Delivery of VEGF and BMPs synergizes to enhance the repair of critical sized-bone defects or ectopic bone formation [13, 14]. FGF2 and BMP2 also showed a synergistic mineralization in cell cultures from elderly mouse and human bone [15]. The synthetic COMP-Ang1 is a soluble variant of Ang1, which is synthesized by replacing the N-terminal portion of Ang1 with the short coiled-coil domain of the COMP. COMP-Ang1 shows potent and stable activity in vascular formation and survival of MSCs and endothelial cells, similar to the endogenous Ang1 [16–18]. Moreover, COMP-Ang1 improves wound healing with increased neovascularization [19]. Interestingly, local delivery of COMP-Ang1 itself accelerates new bone formation in rat calvarial defects [20], suggesting that COMP-Ang1 can be clinically used to treat bone defects, as well as to stimulate bone regeneration. Moreover, COMP-Ang1 synergistically enhances BMP2-induced osteoblastic differentiation through increased Smad1/5/8 phosphorylation [21] and accelerates bone formation during distraction osteogenesis [22]. However, the therapeutic potential of COMP-Ang1 with BMP2 on craniofacial bone defects has not been explored. Combined delivery of COMP-Ang1 and BMP2 is expected to be advantageous for bone repair with angiogenesis via recruitment of MSC-like pericytes and its osteoblastic differentiation.

The aim of this study was to determine the combinatory effects of COMP-Ang1 and BMP2 on cranial bone regeneration with the underlying mechanism and evaluate therapeutic potentials. Our results showed that the combination can enhance the repair of bony defects with the increased pericytes recruitment and their osteogenic differentiation.

## Materials and Methods

### Reagents and recombinant proteins

Recombinant COMP-Ang1 and BMP2 was purchased from Innotherapy (Seoul, Korea) and Cowellmedi (Seoul, Korea), respectively. The absorbable collagen sponge CollaDerm was obtained from Bioland (Ochang, Korea).

### Animal preparations

All animal studies were reviewed and approved by the Animal Ethics Committee of Chonnam National University (No. CNU-IACUC-YB-2014-35). Six week-old male C57BL/6 mice were purchased from Daehan Biolink (Eumseong, Korea) and housed in a housed temperature-controlled condition with 12 h light/dark cycles at Chonnam National University School of Dentistry. Mice were anesthetized by intraperitoneal injection of a mixture of Zoletil (30 mg/kg; Virbac Lab, Carros, France) and Rompun (10 mg/kg; Bayer Korea Ltd, Gyeonggido, Korea). A 0.8 to 1.0-cm sagittal incision was made on the scalp, and then calvarial bone was exposed by blunt dissection. A critical size defect was created by means of a 5-mm inner diameter trephine bur under sterile environment. After BMP2 and/or COMP-Ang1 with absorbable collagen sponges were implanted into the defects, surgical incisions were closed. Animals were sacrificed by carbon dioxide exposure at a low-flow rate of 10%-30% of the cage volume per minute after 3 weeks of surgical treatment, and calvarial bones were harvested for further analysis.

### Micro-computed tomography analysis

Calvarial bone was harvested 3 weeks after implanting, and radiographic analysis for evaluating bone repair was performed with a micro-computed tomography **(**μ**-**CT) apparatus (Skyscan 1172; Skyscan, Aartselaar, Belgium). Each specimen was scanned in a cone-beam acquisition mode. The X-ray source was set at 50 kV and 200 μA with 0.5-mm aluminum filter and at a 17.09 μm resolution. Exposure time was 1.2 sec. From the scan, 449 projections were acquired over an angular range of 180°(angular step; 0.4°), and the image slices were reconstructed with the Nrecon program (version 1.10.0.5, Skyscan). Bone volume repaired was measured by using the CT-Analyzer program (version 1.10.0.5, Skyscan). The 3D surface rendering image was obtained by Mimics imaging program (version 14.0, Materialise N.V., Leuven, Belgium).

### Histology and immunofluorescence analysis

All specimens were decalcified in rapid decalcifying solution (Calci-Clear Rapid, National Diagnostics, Atlanta, GA) for 10 days and then embedded in paraffin, and cut into 7 μm sections. The sections were deparaffinized in xylene, rehydrated with a graded series of alcohols, and stained with hematoxylin and eosin solution.

Immunofluorescence assay was performed to evaluate vascular formation and pericyte recruitment. Briefly, histology sections were deparaffinized, rehydrated, and incubated in phosphate buffered saline solution with Tween-20 (PBS-T), containing 5% normal serum for 2 hrs. After overnight incubation with primary mouse anti-NG2 (1:100; Abcam, Cambridge, UK) and rabbit anti-CD31 (1:50; Abcam, Cambridge, UK) antibodies, the histology sections were treated with Alexa Fluor 488-conjugated donkey anti-mouse IgG and Alexa Fluor 555-conjugated goat anti-rabbit IgG (Santa Cruz Biotechnology, Delaware, CA, USA). Cell nuclei were stained with 4′, 6-Diamidino-2-phenylindole dihydrochloride (DAPI). Immunoreactivity was detected by confocal microscopy (Carl Zeiss, Jena, Germany). The fluorescence intensity was measured from 4 different samples (at least 5 foci per section) by image analyzer LSM 510 software (Carl Zeiss, Jena, Germany).

### Cell culture experiments

The human brain microvascular pericytes (NeuroVascular Coordination Research Center; Seoul, Korea) [23] were cultured in Dulbecco's Modified Eagle's medium (DMEM, Invitrogen, USA) supplemented with 10% fetal bovine serum (FBS, Invitrogen, USA) and 1% antibiotics (Sigma, MO, USA), and incubated at 37°C in a humidified atmosphere of 5% CO_2_. For osteogenic differentiation, 50 μg/ml of ascorbic acid and 5 mM of β-glycerophosphate were added into the culture medium. Cells were maintained for 3 or 15 days in the presence of COMP-Ang1 (600 ng/ml) and/or BMP2 (200 ng/ml), and then harvested for RT-PCR or Alizarin red staining, respectively. Osteogenic medium was replaced every 3 days.

### Cell migration assay

Migration activity of pericytes was evaluated with a Boyden's chamber containing polycarbonate filters with 8-μm pores (Corning, NY, USA) [24]. Cells were seeded onto upper chambers in transwell-24 well culture plates at a density of 5×10^4^ cells/cm^2^ and lower chambers were filled with serum-free DMEM containing COMP-Ang1 (600 ng) and/or BMP2 (200 ng), followed by incubation at 37°C for 6 hrs in a humidified atmosphere of 5% CO_2_. After removing non-migrating cells with a cotton swab, the remaining cells were fixed with 4% paraformaldehyde in PBS and stained with 0.1% crystal violet solution. Cells that had migrated through the transwell membrane pores toward the bottom chamber were counted in 5 random fields from each membrane under a light microscope (Leica, Wetzlar, Germany).

### Reverse transcriptase PCR

Total RNA was isolated using TRIzol reagent (Invitrogen, Carlsbad, CA, USA) according to the manufacturer’s instruction. The cDNA was synthesized with the random primer and reverse transcriptase (Invitrogen). Each reaction consisted of initial denaturation at 94°C for 1 min followed by 3-step cycling: denaturation at 94°C for 30 sec, annealing at a temperature optimized for each primer pair for 30 sec, and extension at 72°C for 30 sec. After the requisite number of cycles (28–30 cycles), the reactions underwent a final extension at 72°C for 5 min. The primer sequences were as follows: human bone sialoprotein (hBSP), forward 5′-AACCTACAACCCCACCACAA-3′ and reverse 5′- AGGTTCCCCGTTCTCACTTT-3′; human osteocalcin (hOCN), forward 5′-CTCACACTCCTCGCCCTATT-3′ and reverse 5′-GCTCCCAGCCATTGATACAG-3′; human osterix (hOSX), forward 5′-GGCACAAAGAAGCCGTACTC-3′ and reverse 5′-TGGGAAAAGGGAGGGTAATC-3′; β-actin, forward 5′- GTCGGGCGCCCCAGGCACCA-3′ and reverse 5′-CTCCTTAATGTCACGCACGAT-3′.

### Western blot analysis

Cells were harvested in a lysis buffer (Cell Signaling, Beverly, MA, USA) and then total protein concentration was determined by the BCA assay reagent (Bio-Rad Laboratories, Hercules, CA, USA). Proteins were resolved on a 10% SDS-PAGE and transferred to a PVDF membrane. After blocking in 5% skim milk in TBS with 0.1% Tween-20 (TBS-T), the membrane was incubated overnight at 4°C with primary antibodies for phospho-Smad1/5/8 and total Smad (Cell Signaling, Beverly, MA, USA) diluted 1:2,000 in 5% skim milk in TBS-T. After washing, the blots were incubated for 2 hrs with anti-rabbit horseradish-peroxidase-conjugated antibody (Promega, Madison, WI, USA) diluted 1:3,000 in TBS-T. Signals were detected by an enhanced chemiluminescence reagent (Santa Cruz Biotech, CA, USA), and LAS-4000 luminoimage analyzer system (Fujifilm, Tokyo, Japan).

### Alizarin red staining

Alizarin Red staining was performed to examine matrix calcification. Briefly, cultured cells were fixed with 70% ethanol for 60 min, rinsed thrice with deionized water, and reacted with a 40 mM alizarin red solution (pH 4.2) for 15 min. The stained cultures were then photographed. For quantitative comparison, stains were extracted with 10% (w/v) cetylpyridinium chloride, and then absorbance was measured with standard alizarin red solution by using spectrophotometry (Multiskan GO, Thermo Scientific, Waltham, USA).

### Statistical analysis

All experiments were repeated at least thrice, and statistical analysis of the data was performed by one-way analysis of variance (ANOVA) and Tukey’s comparisons using the Graph Pad Prism 4 for Windows statistical software package (Graph Pad Software Inc., La Jolla, CA, USA). All data presented were expressed as mean ± SEM of 3 independent measurements. A value of *p* < 0.05 was considered to be statistically significant.

## Results

### Combination of COMP-Ang1 and BMP2 proteins shows enhanced ability to repair critical-sized cranial defects

**Fig 1:**
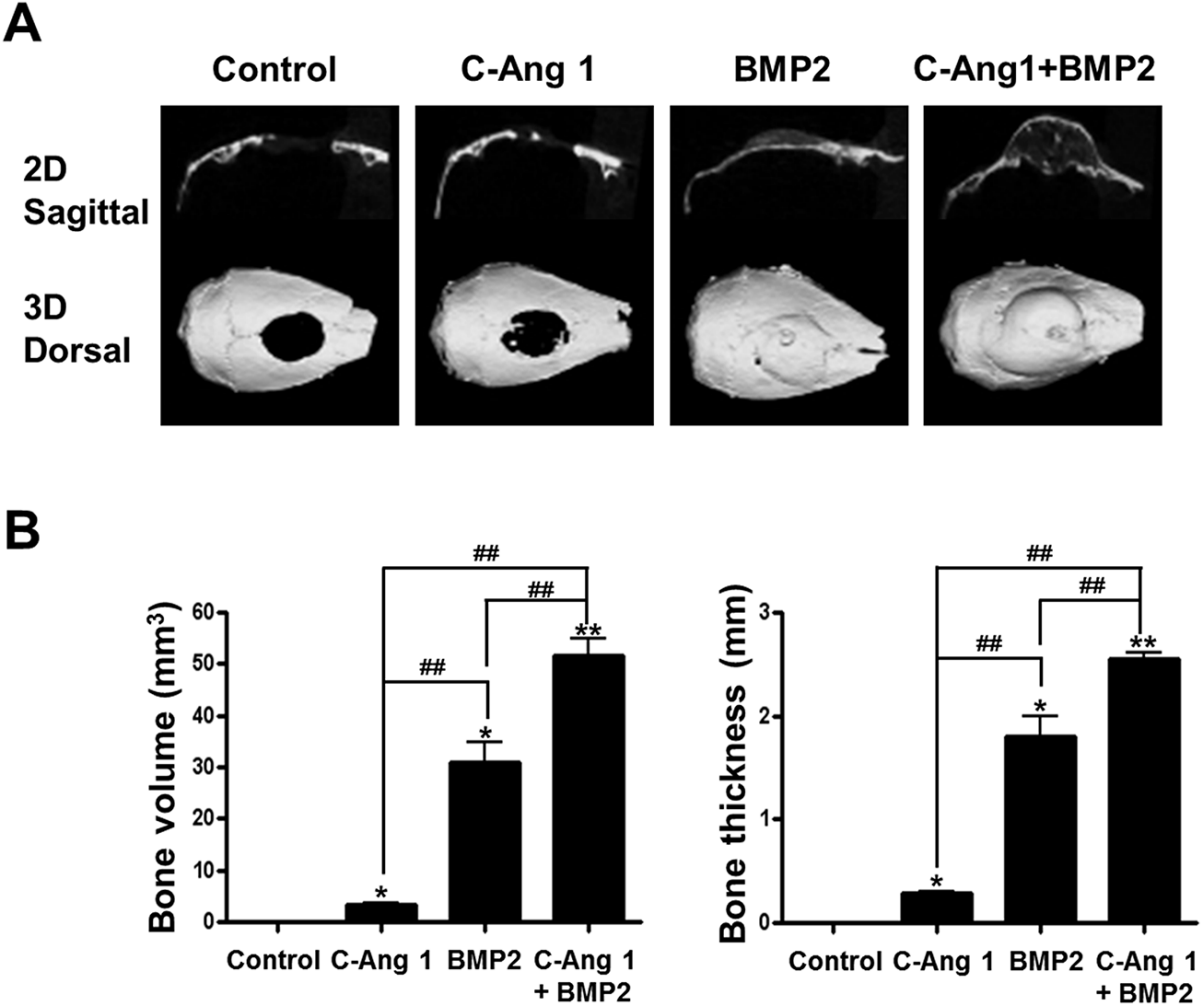
Repair of critical-sized cranial defects by combination of COMP-Ang1 and BMP2. The 5 mm diameter of critical-sized defect was created in the cranium of mice, and COMP-Ang1 (12 μg), BMP2 (4 μg), and COMP-Ang1 (12 μg) plus BMP2 (4 μg) with absorbable collagen sponges were implanted into the defects. Three weeks after surgery, newly formed cranial bone from each group were harvested and analyzed by μ-CT. A, Representative radiographic findings of cranial repairs were shown. Upper panel shows 2-dimensional sagittal views of cranial bone and lower panel shows the dorsal view of 3-dimensionally surface renderings. B, Volume and thickness of regenerated bone in the defects was quantified by CT-Analyzer program. *, *p <*0.05, and **, *p <*0.01 compared to control group, respectively. ^#^, *p <*0.05 and ^##^, *p <*0.01, compared to the indicated group. n = 4.

**Fig 2:**
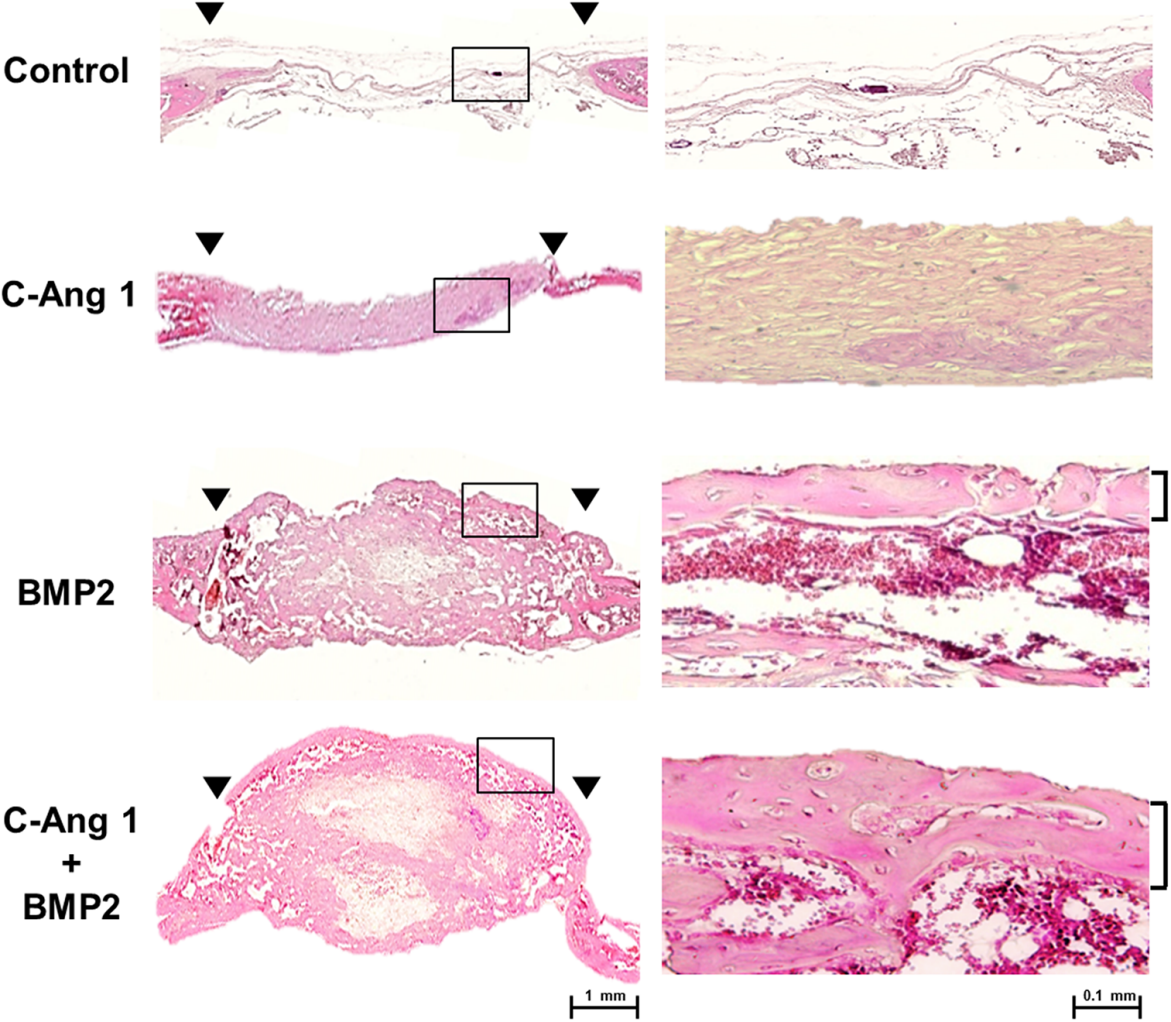
Microphotographs of regenerated cranial bone by COMP-Ang1 or/and BMP2. The samples of radiographic study (Fig 1) were prepared for histology. Sagittal sections through the midline of defects are shown with hematoxylin and eosin stain. Arrowheads in left panel indicate margins of the trephine defect (X40; Bar, 1 mm). Right panel is the magnified images of box areas in left panel (X200; Bar, 0.1 mm).

Previously, we showed that pro-angiogenic COMP-Ang1 could enhance the BMP2-induced ectopic bone formation and osteogenesis [21]. We focused on the combined effect of recombinant COMP-Ang1 and BMP2 proteins on orthotopic bone regeneration in critical-sized cranial defects. COMP-Ang1 (12 μg) and/or BMP2 (4 μg) was transferred into cranial bony defects with absorbable collagen sponges and after 3 weeks bone repair regions were evaluated using by μ-CT analysis. COMP-Ang1 delivery produced marginal healing, as compared with the control group; and BMP2 delivery induced big bone to almost cover the defects. Interestingly, combined delivery of COMP-Ang1 and BMP2 revealed more robust bone formation in the defect regions, as compared to the delivery of BMP2 alone (Fig 1A). When bone volume in defect space was measured, COMP-Ang1 also significantly induced bone formation, as compared with collagen sponge control (3.44 ± 0.21 mm^3^ vs. 0.55 ± 0.15 mm^3^, *p* < 0.05). Combined delivery of COMP-Ang1 and BMP2 produced greater bone by approximately 1.5 times, as compared to the BMP2-treated group (51.72 ± 3.18 mm^3^ vs. 30.99 ± 3.80 mm^3^, *p* < 0.01). According to the new bone thickness parameter, COMP-Ang1 delivery showed the production of thin bone with 0.29 ± 0.02 mm thickness. In the collagen sponge group, thickness of new bone was not measurable. The combined delivery of COMP-Ang1 and BMP2 induced approximately 1.5 times thicker bone, as compared to the BMP2 group (2.56 ± 0.09 mm vs. 1.81 ± 0.32 mm, *p* < 0.01) (Fig 1B). Histology analysis showed that the defect regions with BMP2 delivery were filled with thick fibrous tissue and newly formed bone. Accordingly, combined delivery of COMP-Ang1 and BMP2 produced bigger bones than BMP2 alone within the defect regions. In the collage sponge control group, defect regions were filled with thin and fibrous tissue without lamellar structure of bone tissue (Fig 2). In addition, inflammatory responses were not observed in the defect regions of all groups.

### COMP-Ang1 and BMP2 stimulate bone repairs with the increased vascular formation and pericyte recruitment

**Fig 3:**
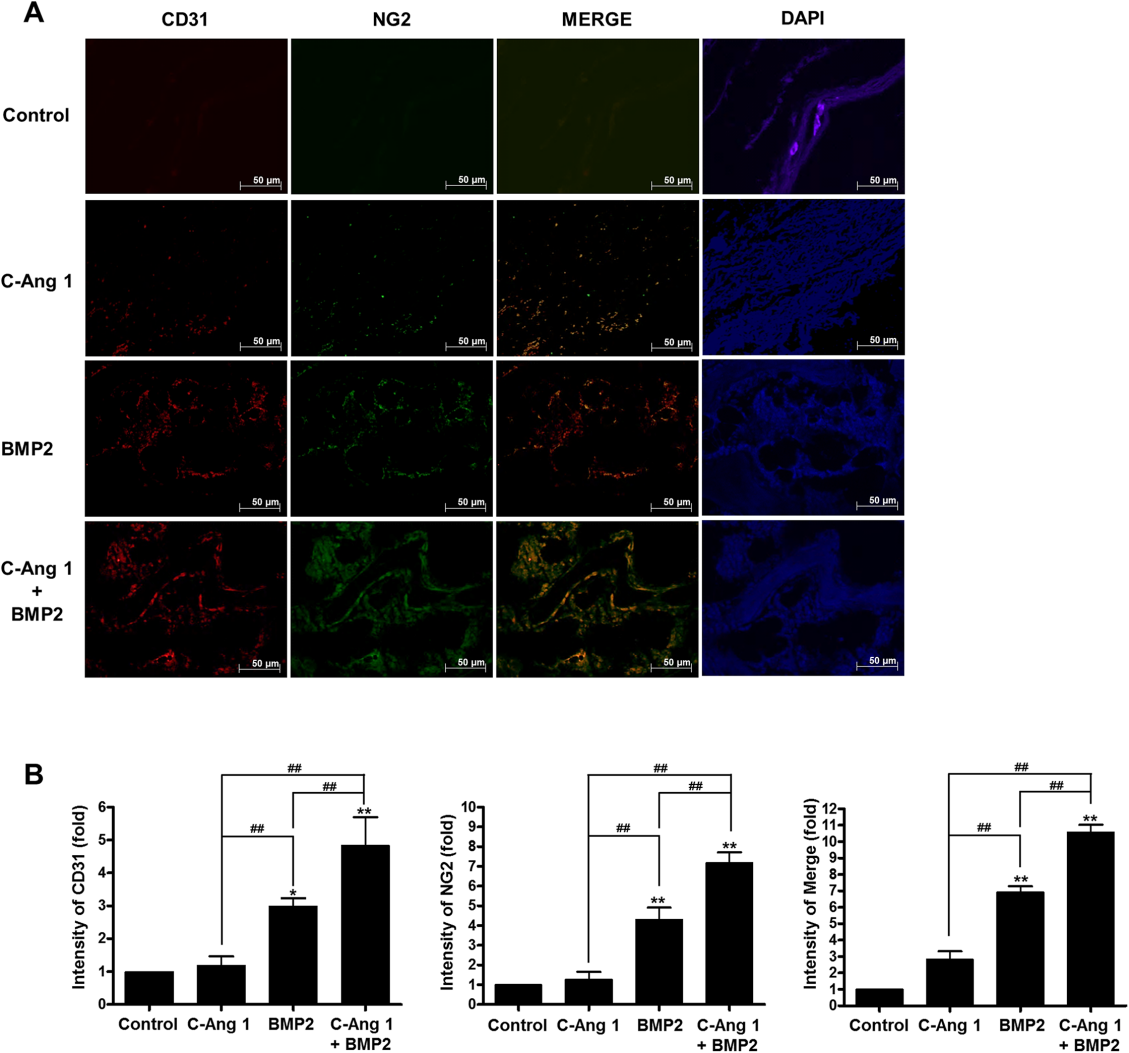
Immunofluorescent analysis for vascular formation and pericyte recruitment in defected regions. (A) Representative images of immunofluorescence staining with primary anti-CD31 and anti-NG2 antibodies. Immunoreactivities for CD31 (a marker of endothelial cells, red) or NG2 (a specific marker of pericyte, green) were visualized by Alexa Fluor 488-conjugated donkey anti-mouse IgG and Alexa Fluor 555-conjugated goat anti-rabbit IgG. For co-localization of endothelial cells and pericytes, the immunofluorescence images were merged (orange). DAPI (blue) was used as a nuclear counterstain. (B) Fold changes of immunofluorescence intensity. Immunoreactivities against anti-CD31 and anti-NG2 were quantified by using image analysis software (Carl Zeiss LSM software). *, *p<*0.05, and **, *p <*0.01 compared to control group, respectively. ^##^, *p <*0.01, as compared to the indicated group. n = 4.

Ang1 acts as a pro-angiogenic factor that induces new vasculature by promoting recruitment of pericytes and vascular smooth muscle cells [19]. Therefore, we examined the effects of COMP-Ang1 and BMP2 on vascular formation and pericytes recruitment with bone repairs using immunofluorescence analysis. Delivery of BMP2 or COMP-Ang1 increased the immunoreactivities against CD31 (a marker of endothelial cells, red) and NG2 (a specific marker of pericyte, green) antibody in the cranial defects, whereas collagen sponge control showed a small populations of CD31- and NG2-positive cells. Combined delivery of COMP-Ang1 and BMP2 produced increased immunoreactivity against CD31 or NG2 antibody. Double-positive reactivity (orange color) against CD31 and NG2 antibodies was also increased by the combination treatment (Fig 3A). Fold changes of fluorescence intensity were presented in Fig 3B; for CD31, 1.18 ± 0.62, 2.99 ± 0.54, and 4.74 ± 0.86 in COMP-Ang1, BMP2, and COMP-Ang1/BMP2, respectively (*p* < 0.01); for NG2, 1.35 ± 0.84, 4.31 ± 1.33, and 7.56 ± 1.18 (*p* < 0.01); for merging, 2.84 ± 1.10, 6.97 ± 0.79, 10.95 ± 0.99, respectively (*p* < 0.01).These results suggested that the enhanced effects of COMP-Ang1 and BMP2 on bone repair might be related to increased angiogenesis and pericyte recruitment.

### Combination of COMP-Ang1 and BMP2 stimulate *in vitro* migration of pericytes and their osteoblastic differentiation

**Fig 4:**
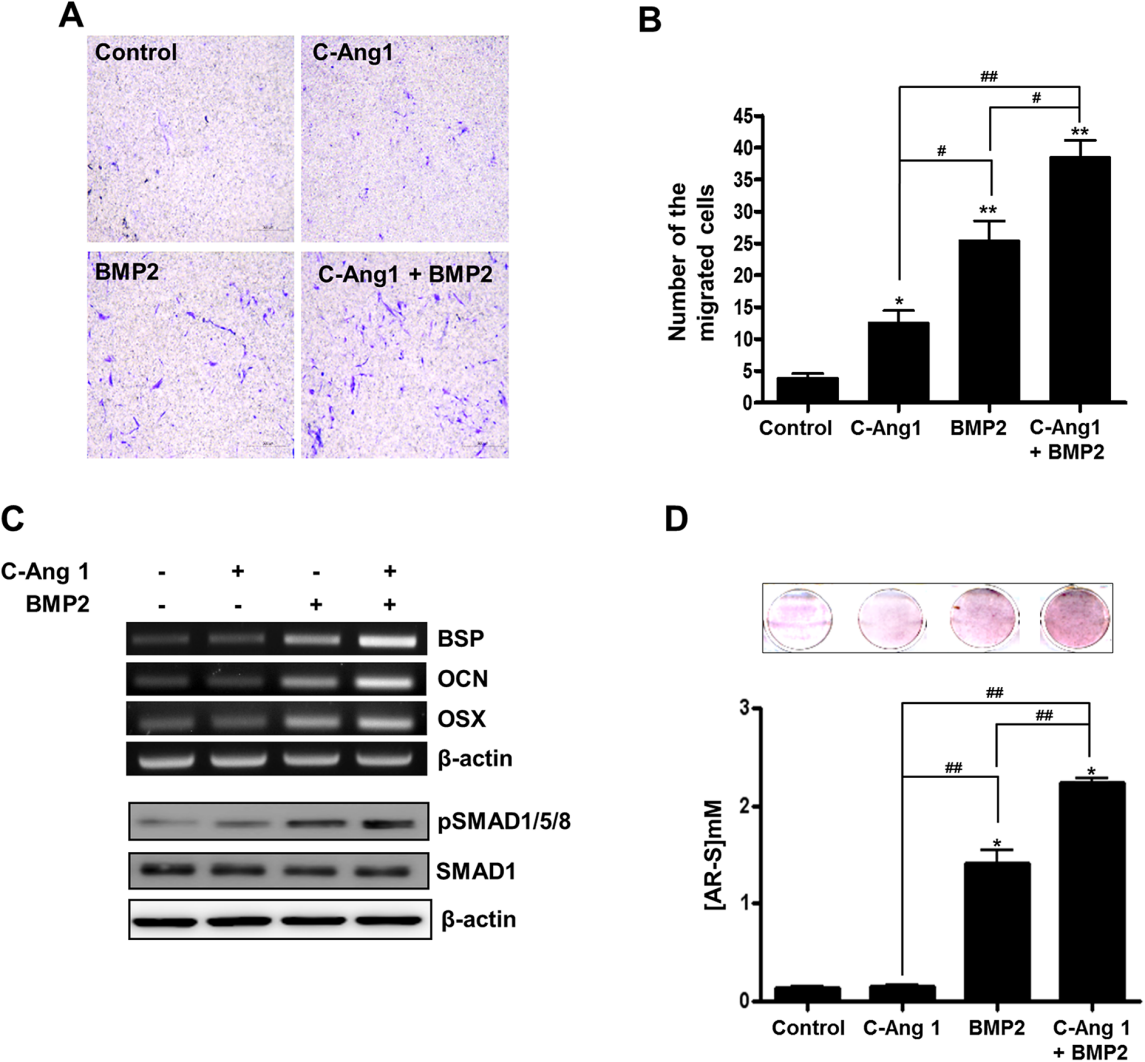
Effects of COMP-Ang1 and BMP2 on migration and osteogenic differentiation of pericytes. (A, B) Migration assay. Human microvascular pericytes were seeded in a transwell chamber with 8-μm pores and maintained with DMEM containing COMP-Ang1 (600 ng/ml) and/or BMP2 (200 ng/ml). After 6 hours, infiltrated cells were fixed with 4% paraformaldehyde and stained with 0.1% crystal violet solution, and then counted under a light microscope. (C) Cells were cultured with DMEM containing 50 μg/ml of ascorbic acid and 5 mM of β-glycerophosphate in the presence of COMP-Ang1 (600 ng/ml) and/or BMP2 (200 ng/ml). After 3 days, cells were harvested and RT-PCR (upper panel) was performed with specific primers for bone sialoprotein, osteocalcin, osterix and β-actin. For Western blot analysis (lower panel), cells were harvested 1 hour after COMP-Ang1 and BMP2 treatment. (D) Calcium deposition assay. Human microvascular pericytes were cultured as in C. After 15 days, cells were stained with alizarin red solution, and then scanned (upper panel). For quantification, the stain was eluted with 10% cetylpyridinium and absorbance was measured by spectrophotometry. *, *p<*0.05, and **, *p <*0.01, as compared to control group, respectively. ^#^, *p<*0.05 and ^##^, *p <*0.01, as compared to the indicated group. n = 3.

Pericyte is a specialized perivascular cell, which is capable of differentiating into a variety of different cell types [25]. Moreover, COMP-Ang1 was previously shown to mediate progenitor cell migration [26]. Therefore, we investigated the combined effects of COMP-Ang1 and BMP2 on cell migration in pericytes *in vitro*. Boyden's chamber assay showed that transwell migration of pericytes was increased by COMP-Ang1 (13 ± 4.77 cells, *p* < 0.05), BMP2 (26 ± 3.65 cells, *p* < 0.01), and their combination (39 ± 4.87 cells, *p* < 0.01), as compared with the control group (4 ± 2.07 cells) (Fig 4A and 4B). To examine the effects of the proteins on osteoblastic differentiation of pericytes, RT-PCR, Western blotting, and alizarin red staining were performed. BMP2 treatment stimulated Smad1/5/8 phosphorylation and osteoblast-specific gene expression such as BSP, OCN, and OSX in the pericytes cultured with osteogenic medium. Furthermore, addition of COMP-Ang1 enhanced BMP2-induced gene expressions and Smad1/5/8 phosphorylation (Fig 4C). Results showed that COMP-Ang1 alone did not affect matrix calcification, as compared to control (COMP-Ang1 vs. control, 0.15 ± 0.03 vs. 0.14 ± 0.02 mM, *p* < 0.05). However, BMP2 treatment induced matrix calcification in pericyte cultures, and addition of COMP-Ang1 synergistically enhanced the BMP2-induced matrix calcification (BMP2 vs. COMP-Ang1/BMP2, 1.41 ± 0.24 vs. 2.23 ± 0.10 mM, *p* < 0.05) (Fig 4D). These results suggested that COMP-Ang1 would enhance BMP2 induction of pericyte migration and osteoblastic differentiation.

## Discussion

In this study, combined delivery of COMP-Ang1 and BMP-2 enhanced bone regeneration of calvarial defects through the increased recruitment and osteogenic differentiation of pericytes. Previously, we observed that COMP-Ang1 enhanced the osteogenesis and ectopic bone formation in synergy with BMP-2 *in vitro* and *in vivo* [21]. The rationale of this study was based on the evidence that BMP-2 can directly induce MSC differentiation to stimulate bone formation and COMP-Ang1 could indirectly take part in bone formation through inducing a network of blood vessels within the defect accompanied by pericyte recruitment.

Perivascular pericytes are a kind of pluripotent MSC-like cell that differentiate into osteoblasts and myoblasts, and adipocytes [4]. In fact, when Anxa5-positive pericytes were exposed to osteogenic medium, calcium deposits were induced with type I collagen expression in the cell culture [5]. BMPs can stimulate osteoblast differentiation of MSC and induce ectopic and orthotopic bone formation in various animal models [11, 27]. In the present study, BMP2 treatment induced osteoblast-specific gene expression, including BSP, OCN and OSX mRNA, and also increased matrix calcification in pericyte cultures. In addition, BMP2 increased the level of phosphorylated Smad1/5/8, a major signal molecule for osteoblast differentiation, within pericytes (Fig 4). These findings confirmed that pericytes are one of the MSCs lineages that are capable of differentiating into osteoblasts in response to BMP2.

Angiogenesis is coupled with osteogenesis in the body and serves the osteoprogenitor cells or MSCs for osteogenesis. In green fluorescent protein-transgenic bone marrow cells transplanted mice, a BMP2-containing collagen pellet induced ectopic bone formation with a significant number of GFP-positive osteoblasts, suggesting bone marrow-derived osteoblast progenitors in circulating blood contribute to ectopic bone formation [28]. The MSC-like pericytes are recruited along the vessel wall into osteogenesis region, and Ang1 is involved in the ingrowth of blood vessel with pericytes into the wound site [4, 10]. In the present study, COMP-Ang1 delivery increased the population of CD31 and NG2 positive cells simultaneously, indicating that COMP-Ang1 induces vascular formation with pericyte recruitment in the target regions. Interestingly, BMP2 delivery itself also induced bone formation with the increased CD31 and NG2 expression within the target regions (Fig 3). These findings suggested that BMP2-induced bone formation is related with the increased angiogenesis and MSCs recruitment via vascular wall. Boyden chamber study (Fig 4) further supported the findings; BMP2 increased pericyte migration into membrane pores.

Combination of angiogenic factors and BMPs enhanced bone formation and osteoblast differentiation in several studies [13–15]. For example, VEGF enhances healing of critical-sized calvarial defects elicited by muscle-derived stem cells expressing BMP2 through modulation of angiogenesis, while sFlt1, a VEGF antagonist, inhibits the bone formation [13]. The localized release of VEGF and BMP2 promote bone regeneration by facilitating bone marrow stem cell homing and differentiation [14]. The synergy could be explained with the different action modes of both factors; VEGF induces blood vessel formation to increase the supply of MSC cells through blood stream or vessel wall, and BMP2 potently differentiates them into osteoblasts. In this study, we examined the effects of another angiogenic COMP-Ang1 on BMP2-induced bone formation and pericyte recruitment. COMP-Ang1 delivery slightly increased healing of critical-sized calvarial defects with mild increases in CD31 and NG2 expression, and BMP2 delivery strongly induced bone healing and CD31 and NG2 expression, as compared with COMP-Ang1, suggesting that BMP2 is also a strong inducer of angiogenesis and MSCs differentiation. Co-delivery of BMP2 and COMP-Ang1 enhanced the bone regeneration with the increases in CD31 and NG2 expressions *in vivo*, and also enhanced migration, osteoblast specific gene expressions and matrix mineralization in pericyte cultures. These findings suggested that COMP-Ang1-induced enhancement of bone formation is due to increased pericyte recruitment

Significant steps have already been made toward the use of recombinant COMP-Ang1 for tissue engineering purposes, because COMP-Ang1 stimulates proliferation, osteoblast differentiation, or migration of bone marrow-MSCs and human periodontal ligament stem cells [26]. For example, COMP-Ang1 delivery in distracted limb and spinal area of rat has shown significant efficacy in the rate of fusion and degree of bone formation [22, 29]. Use of COMP-Ang1 has several potential advantages, as compared to natural Ang1 and VEGF, including efficiency of generation of recombinant protein, potency, and Tie2 activation [16]. Our study showed that co-delivery of COMP-Ang1 and BMP2 would be a good strategy for bone tissue regeneration.

For successful tissue regeneration, an appropriate carrier for specific molecules is needed so as to retain them at the delivery site for a sufficient time [30]. In the present study, a commercial absorbable collagen sponge was used for delivering COMP-Ang1 or BMP2. The delivery of COMP-Ang1 with the carrier slightly induced marginal bone regeneration of calvarial defects. The carrier alone did not produce any new bone. However, a recent study showed that local delivery of COMP-Ang1 without BMP2 also regenerated new bones, which are almost covered over calvarial defects in rat [20]. In the study, a modified carrier of type I atelocollagen was used for COMP-Ang1 delivery, and the carrier alone also induced new bone. The discrepancy of COMP-Ang1 effects on calvarial bone regeneration appears to be due to the type of carrier used. These findings suggested that releasing kinetics of COMP-Ang1 or BMP2 and their biological activities could be variable, depending on the type of carrier used. To achieve the most effective bone regeneration with COMP-Ang1 or BMP2, further studies are required to identify an optimal carrier for controlling release initiation, period of release, and ratio of these molecules.

Angiogenesis and pericyte migration into target regions might be followed by detachment of pericytes from existing basal membrane/pericyte complex or stable vasculature [31, 32]. In the present study, we could not observe directly detachment of pericytes and migration into bony defects. However, we cannot absolutely exclude the detachment and migration of pericyte for angiogenesis and osteogenesis, because the immunostaining analysis could be performed with low-resolution of microscope after pericyte-relocating to basal membrane or endothelial cells. The molecular mechanism by which COMP-Ang1 and BMP2 control the detachment and migration of pericytes still remains to be determined in further study.

In conclusion, co-delivery of COMP-Ang1 and BMP2 can enhance bone repairs in cranial defects. COMP-Ang1 might mainly serve as conduit to recruit pericytes for bone regeneration, whereas, BMP2 might stimulate both migration and osteogenic differentiation of pericytes to construct the repairing bone. These results may be helpful to develop therapeutic strategies for controlling the defected bones and repairs.

## References

[1] Chim SM, Tickner J, Chow ST, Kuek V, Guo B, Zhang G, et al Angiogenic factors in bone local environment. Cytokine Growth Factor Rev 2013; 24: 297–310. 10.1016/j.cytogfr.2013.03.008 23611723

[2] Yu YY, Lieu S, Lu C, Colnot C. Bone morphogenetic protein 2 stimulates endochondral ossification by regulating periosteal cell fate during bone repair. Bone 2010; 47: 65–73. 10.1016/j.bone.2010.03.012 20348041 PMC2891074

[3] Athanasopoulos AN, Schneider D, Keiper T, Alt V, Pendurthi UR, Liegibel UM, et al Vascular endothelial growth factor (VEGF)-induced up-regulation of CCN1 in osteoblasts mediates proangiogenic activities in endothelial cells and promotes fracture healing. J Biol Chem 2007; 282: 26746–26753. . 17626014 10.1074/jbc.M705200200 PMC2831223

[4] Bautch VL. Stem cells and the vasculature. Nat Med 2011; 17: 1437–43. 10.1038/nm.2539 22064433

[5] Brachvogel B, Moch H, Pausch F, Schlotzer-Schrehardt U, Hofmann C, Hallmann R, et al Perivascular cells expressing annexin A5 define a novel mesenchymal stem cell-like population with the capacity to differentiate into multiple mesenchymal lineages. Development 2005; 132: 2657–2668. . 15857912 10.1242/dev.01846

[6] Crisan M, Yap S, Casteilla L, Chen CW, Corselli M, Park TS, et al A perivascular origin for mesenchymal stem cells in multiple human organs. Cell Stem Cell 2008; 3: 301–313. 10.1016/j.stem.2008.07.003 18786417

[7] Gerhardt H, Betsholtz C. Endothelial-pericyte interactions in angiogenesis. Cell Tissue Res 2003; 314: 15–23. . 12883993 10.1007/s00441-003-0745-x

[8] Stratman AN, Schwindt AE, Malotte KM, Davis GE. Endothelial-derived PDGF-BB and HB-EGF coordinately regulate pericyte recruitment during vasculogenic tube assembly and stabilization. Blood 2010; 116: 4720–4730. 10.1182/blood-2010-05-286872 20739660 PMC2996127

[9] Hoeben A, Landuyt B, Highley MS, Wildiers H, Van Oosterom AT, De Bruijn EA. Vascular endothelial growth factor and angiogenesis. Pharmacol Rev 2004; 56: 549–580. . 15602010 10.1124/pr.56.4.3

[10] Cai J, Kehoe O, Smith GM, Hykin P, Boulton ME. The angiopoietin/tie-2 system regulates pericyte survival and recruitment in diabetic retinopathy. Invest Ophthalmol Vis Sci 2008; 49: 2163–2171. 10.1167/iovs.07-1206 18436850

[11] Franceschi RT. Biological approaches to bone regeneration by gene therapy. J Dent Res 2005; 84: 1093–1103. . 16304438 10.1177/154405910508401204

[12] Duguy N, Petite H, Arnaud E. Biomaterials and osseous regeneration. Ann Chir Plast Esthet 2000; 45: 364–376. . 10929463

[13] Peng H, Usas A, Olshanski A, Ho AM, Gearhart B, Cooper GM, et al VEGF improves, whereas sFlt1 inhibits, BMP2-induced bone formation and bone healing through modulation of angiogenesis. J Bone Miner Res 2005; 20: 2017–2027. . 16234975 10.1359/JBMR.050708

[14] Zhang W, Zhu C, Wu Y, Ye D, Wang S, Zou D, et al VEGF and BMP-2 promote bone regeneration by facilitating bone marrow stem cell homing and differentiation. Eur Cell Mater 2014; 27: 1–11. . 24425156 10.22203/ecm.v027a01

[15] Kuhn LT, Ou GM, Charles L, Hurley MM, Rodner CM, Gronowicz G. Fibroblast growth factor-2 and bone morphogenetic protein-2 have a synergistic stimulatory effect on bone formation in cell cultures from elderly mouse and human bone. J Gerontol A Biol Sci Med Sci 2013; 68: 1170–1180. 10.1093/gerona/glt018 23531867 PMC3826858

[16] Cho CH, Kammerer RA, Lee HJ, Steinmetz MO, Ryu YS, Lee SH, et al COMP-Ang1: a designed angiopoietin-1 variant with nonleaky angiogenic activity. Proc Natl Acad Sci USA 2004; 101: 5547–5552. . 15060279 10.1073/pnas.0307574101 PMC397420

[17] Kim I, Kim HG, Moon SO, Chae SW, So JN, Koh KN, et al Angiopoietin-1 induces endothelial cell sprouting through the activation of focal adhesion kinase and plasmin secretion. Circ Res 2000; 86: 952–959. . 10807867 10.1161/01.res.86.9.952

[18] Lee KN, Seo MC, Bae IH, Oh SH, Jang WG, Jeong BC, et al COMP-Ang1, a variant of angiopoietin 1, inhibits serum-deprived apoptosis of mesenchymal cells via PI3K/Akt and mitogen-activated protein kinase pathways. Pharmacology 2010; 86: 327–335. 10.1159/000321885 21109762

[19] Koh GY. Orchestral actions of angiopoietin-1 in vascular regeneration. Trends Mol Med 2013; 19: 31–39. 10.1016/j.molmed.2012.10.010 23182855

[20] Lim SS, Kook SH, Bhattarai G, Cho ES, Seo YK, Lee JC. Local delivery of COMP-angiopoietin 1 accelerates new bone formation in rat calvarial defects. J Biomed Mater Res A 2015; 103: 2942–2951. 10.1002/jbm.a.35439 25727390

[21] Jeong BC, Kim HJ, Bae IH, Lee KN, Lee KY, Oh WM, et al COMP-Ang1, a chimeric form of Angiopoietin 1, enhances BMP2-induced osteoblast differentiation and bone formation. Bone 2010; 46: 479–486. 10.1016/j.bone.2009.09.019 19782780

[22] Park BH, Yoon SJ, Jang KY, Kim MR, Lee HS, Kim KB, et al COMP-angiopoietin-1 accelerates bone formation during distraction osteogenesis. Bone 2010; 46: 1442–1448. 10.1016/j.bone.2010.02.004 20149905

[23] Lee HJ, Ahn BJ, Shin MW, Jeong JW, Kim JH, Kim KW. Ninjurin1 mediates macrophage-induced programmed cell death during early ocular development. Cell Death Differ 2009; 16: 1395–1407. 10.1038/cdd.2009.78 19557008

[24] Kwon JS, Kim YS, Cho AS, Cho HH, Kim JS, Hong MH, et al The novel role of mast cells in the microenvironment of acute myocardial infarction. J Mol Cell Cardiol 2011; 50: 814–825. 10.1016/j.yjmcc.2011.01.019 21295578

[25] Collett GD, Canfield AE. Angiogenesis and pericytes in the initiation of ectopic calcification. Circ Res 2005; 96: 930–938. . 15890980 10.1161/01.RES.0000163634.51301.0d

[26] Kook SH, Lim SS, Cho ES, Lee YH, Han SK, Lee KY, et al COMP-angiopoietin 1 increases proliferation, differentiation, and migration of stem-like cells through Tie-2-mediated activation of p38 MAPK and PI3K/Akt signal transduction pathways. Biochem Biophys Res Commun 2014; 455: 371–377. 10.1016/j.bbrc.2014.11.025 25446117

[27] Garrison KR, Donell S, Ryder J, Shemilt I, Mugford M, Harvey I, et al Clinical effectiveness and cost-effectiveness of bone morphogenetic proteins in the non-healing of fractures and spinal fusion: a systematic review. Health Technol Assess 2007; 11: 1–150. . 17669279 10.3310/hta11300

[28] Otsuru S, Tamai K, Yamazaki T, Yoshikawa H, Kaneda Y. Bone marrow-derived osteoblast progenitor cells in circulating blood contribute to ectopic bone formation in mice. Biochem Biophys Res Commun 2007; 354: 453–458. . 17239347 10.1016/j.bbrc.2006.12.226

[29] Park BH, Song KJ, Yoon SJ, Park HS, Jang KY, Zhou L, et al Acceleration of spinal fusion using COMP-angiopoietin 1 with allografting in a rat model. Bone 2011; 49: 447–454. 10.1016/j.bone.2011.05.020 21658484

[30] Vo TN, Kasper FK, Mikos AG. Strategies for controlled delivery of growth factors and cells for bone regeneration. Adv Drug Deliv Rev 2012; 64: 1292–1309. 10.1016/j.addr.2012.01.016 22342771 PMC3358582

[31] Ozerdem U, Stallcup WB. Early contribution of pericytes to angiogenic sprouting and tube formation. Angiogenesis 2003; 6: 241–249. . 15041800 10.1023/B:AGEN.0000021401.58039.a9 PMC1371062

[32] Schrimpf C, Teebken OE, Wilhelmi M, Duffield JS. The role of pericyte detachment in vascular rarefaction. J Vasc Res 2014; 51: 247–258. 10.1159/000365149 25195856 PMC4476411

